# Special Issue “Transmission Dynamics of Insect Viruses”

**DOI:** 10.3390/v12060644

**Published:** 2020-06-14

**Authors:** Kenneth A. Stapleford

**Affiliations:** Department of Microbiology, New York University School of Medicine, New York, NY 10016, USA; kenneth.stapleford@nyulangone.org

At the close of this Special Issue of *Viruses* on the Transmission Dynamics of Insect Viruses, we would like to thank all of the authors for their submissions and the great work expanding our knowledge of insect virus biology and transmission.

We are pleased with the breadth of high-quality research articles touching on multiple areas of biology. We have learned of the detection of the Reo-Like virus in Brazil [1], the Toscana virus in France [2], the transmission cycles in the Amazon [3], and how Zika virus can be transmitted by mechanical means [4]. These articles have significantly added to our knowledge of how insect viruses are detected and transmitted in nature. Moreover, articles focusing on alphavirus fidelity [5], the role of macropinocytic vesicles in nucleopolyhedrovirus infection [6], and Insect small RNA responses to dengue virus and West Nile virus [7,8] have expanded our understanding of the complex host-pathogen interactions taking place during insect virus infections. 

In addition, we have published thorough reviews highlighting the roles of temperature [9], RNA interference [10], vector competence [11], codon usage [12], and mosquito-specific [13] and emerging insect virus pathogens [14]. These relevant and timely reviews have nicely complemented the research articles of the issue and opened our eyes to many of the pressing questions that still need to be addressed in the field.

With summer upon us and insects making themselves known, it is hard not to think of insect virus transmission and disease. These articles and reviews have helped us to understand a bit more of how insect viruses are transmitted and have filled some of the gaps in our knowledge regarding the transmission dynamics of insect viruses. We look forward to future works from many investigators to bring us closer to understanding the complex and exciting relationship between insects and viruses.
